# Catalytic Pyrolysis Kinetic Behavior and TG-FTIR-GC–MS Analysis of Metallized Food Packaging Plastics with Different Concentrations of ZSM-5 Zeolite Catalyst

**DOI:** 10.3390/polym13050702

**Published:** 2021-02-26

**Authors:** Justas Eimontas, Nerijus Striūgas, Mohammed Ali Abdelnaby, Samy Yousef

**Affiliations:** 1 Laboratory of Combustion Processes, Lithuanian Energy Institute, Breslaujos 3, LT-44403 Kaunas, Lithuania; Justas.Eimontas@lei.lt (J.E.); Nerijus.Striugas@lei.lt (N.S.); 2 Department of Production Engineering and Printing Technology, Akhbar Elyom Academy, Cairo 12566, Egypt; Muhmmad.aly@akhbaracademy.edu.eg; 3 Department of Production Engineering, Faculty of Mechanical Engineering and Design, Kaunas University of Technology, LT-51424 Kaunas, Lithuania; 4 Department of Materials Science, South Ural State University, Lenin Prospect 76, 454080 Chelyabinsk, Russia

**Keywords:** metallized food packaging plastics waste, catalytic pyrolysis, ZSM-5 Zeolite catalyst, TG-FTIR-GC–MS analysis, pyrolysis kinetic behavior

## Abstract

Recently, the pyrolysis process has been adapted as a sustainable strategy to convert metallized food packaging plastics waste (MFPW) into energy products (paraffin wax, biogas, and carbon black particles) and to recover aluminum. Usually, catalysts are used in pyrolysis treatment to refine pyrolysis products and to increase their yield. In order to study the effect of a catalyst on the formulated volatile products, this work aims to study the pyrolysis behavior of MFPW in presence of catalyst, using TG-FTIR-GC–MS system. The pyrolysis experiments were conducted with ZSM-5 Zeolite catalyst with different concentrations (10, 30, and 50 wt.%) at different heating rates (5, 10, 15, 20, 25, and 30 °C/min). In addition, TG-FTIR system and GC-MS unit were used to observe and analyze the thermal and chemical degradation of the obtained volatile compounds at maximum decomposition peaks. In addition, the kinetic results of catalytic pyrolysis of ZSM-5/MFPW samples matched when model-free methods, a distributed activation energy model (DAEM), and an independent parallel reaction kinetic model (IPR) were used. The TGA-DTG results showed that addition of a catalyst did not have a significant effect on the features of the TGA-DTG curves with similar weight loss of 87–90 wt.% (without taking the weight of the catalyst into account). Meanwhile, FTIR results manifested strong presence of methane and high-intensity functional group of carboxylic acid residues, especially at high concentration of ZSM-5 and high heating rates. Likewise, GC-MS measurements showed that Benzene, Toluene, Hexane, p-Xylene, etc. compounds (main flammable liquid compounds in petroleum oil) generated catalysts exceeding 50%. Finally, pyrolysis kinetics showed that the whole activation energies of catalytic pyrolysis process of MFPW were estimated at 289 kJ/mol and 110, 350, and 174 kJ/mol for ZSM-5/MFPW samples (10, 30, and 50 wt.%, respectively), whereas DAEM and IPR approaches succeeded to simulate TGA and DTG profiles with deviations below <1.

## 1. Introduction

Thermal treatments are among the most common practices used to treat the millions of plastic wastes generated annually and turn them into energy products [1]. This type of practice includes three basic types: incineration, gasification, and pyrolysis process [2]. The products resulting from treating plastic waste vary between thermal energy used in heating systems during winter time, and char in the form of carbon black that can be used as a solid fuel, biogas, oil, wax, etc., [3,4,5]. When compared with all these energy products, pyrolysis oil product has a higher calorific value and better economic performance [6,7]. In addition, the pyrolysis treatment is considered to be the closest technique to reality, even if chemical and mechanical treatments are characterized by high yield and good economic performance, [8,9,10]. Therefore, many authors have studied valorization of plastic waste using pyrolysis treatment. These studies were focused on finding the optimum conditions, in which the maximum yield of oil could be achieved, and these conditions are pyrolysis temperature, pyrolysis time, heating rate, and nitrogen flow rate [11,12]. Additionally, it was observed that due to different composition, each type of plastic waste needs a different pyrolysis conditions [13]. Thus, pyrolysis of plastic waste can be divided into the following main categories: pure plastic waste and metallized plastic waste [14]. Pure plastic waste is polymeric waste composed from polymer component (e.g., mechanical components, spine, etc.) or it has only layers made from bags, packages, etc., [15]. This kind of waste can decompose during the pyrolysis treatment into oil and small amount of char. Meanwhile, the second category (metallized plastic waste) is defined as single or multi polymeric layers coated or joined with metal layer (metallized food packaging plastics waste (MFPW)) with high environmental impact [16,17]. Although this category, including (MFPW), is rich in volatile matter (up to 99.5 wt.%), it is classified as the most complex part of plastic waste with poor recycling rate <20% because of its complex structure [18].

Despite the complexity of MFPW, the thermogravimetric analysis showed that all these layers decompose together in the form of single reaction and can simulate their pyrolysis kinetic parameters using the model-free methods [19], while the upscaling pyrolysis process succeeded in converting it into wax, gas, and char mixed with metal fraction (alumina element) [20]. In addition, char can be used as a filler in fiberglass/epoxy composites after refining it with the help of chemical treatment [21]. Although the results are promising in terms of conversion rate, smaller emission, and better economic performance, the whole volatile matter content was not extracted in the optimal form, in particular oil. Usually, the catalytic pyrolysis process is used for that purpose, to upgrade the products and to add different catalysts during the reaction [22,23]. In the literature, there are many types of catalysts used with plastic solid waste, such as ZSM-5 Zeolite, Red Mud, Y-Zeolite, Natural Zeolite, H-Y Zeolite, Na_2_CO_3_, FCC [24,25]. Among all these types of catalysts, ZSM-5 showed the highest yield of oil reaching 70%. In addition, it was noticed in the studies that the concentration of ZSM-5 has significant impact on the yield [26,27].

However, the selection of ZSM-5 concentration (catalyst to feedstock ratio) was a point of contention among researchers and the evidence for this is the big difference in range of catalysts from 10% to 50%. Due to economic considerations, it is difficult to determine the optimal concentrations that may be achieved by the maximum volatile compounds in a pilot reactor. Thus, thermogravimetry is the best tool for that purpose. In addition, thermogravimetric data help the best to determine the kinetic parameters of MFPWs, which are very useful for upscaling and design of thermal reactors and their geometry (thickness, diameter, etc.) and materials [28]. Actually, the pyrolysis kinetic of MFPW was studied using model-free and independent parallel reaction (IPR) [19,29], while the catalytic pyrolysis kinetic of MFPW and the effect of the concentration of catalyst on their compounds are still missing. Within this frame and in order to better understand the catalytic pyrolysis kinetic behavior of MFPW, this work aims to study the catalytic pyrolysis of MFPW in presence of ZSM-5 Zeolite catalyst with different loads (10, 30, and 50 wt.%), using TG-FTIR-GC–MS system. In addition, the catalytic pyrolysis kinetic behavior of MFPW and TGA-DTG curves were simulated by Kissinger–Akahira–Sunose, Flynn–Wall–Ozawa, and Friedman using the distributed activation energy model (DAEM) and IPR.

## 2. Experimental

### 2.1. Materials and Feedstock Selection and Preparation

Pyrolysis experiments using TGA were conducted with a mixture from five packaging products: potato chips, chocolate, bakery products, coffee, and biscuits. The food products were purchased from a local shop in Vilnius, Lithuania. The packaging was removed from the products, cut into small pieces (5 mm × 15 mm), brewed in warm water for 5 min, and then thoroughly washed and left for drying overnight. After that, the dried samples were mixed in equal shares, and the mixed batch was milled into fine particles estimated at 200 μm using a coffee grinder for 5 min. Based on our previous study, the milled sample was composed mainly of 84% of polymeric part (PET, LDPE, and EVA) and 16% Al. Additionally, elemental, proximate, and composition content of the sample was estimated at 82.24 (C), 14.07 (H), 0.45 (N), 0.018 (S) 3.21 (O), 0.25 (Moisture), 90.652 (Volatile Matter), 7.49 (Fixed Carbon), and 1.60 (Ash) [20]. Measurements were repeated three times, and then the average of the calculated values was taken. Finally, all the consumed chemicals and ZSM-5 Zeolite catalyst used for the research were purchased from Sigma-12 Aldrich Corp (Kaunas, Lithuania), while gases were provided by the Lithuanian Energy Institute (Kaunas, Lithuania).

### 2.2. Design of the Research Experiments

**Figure 1:**
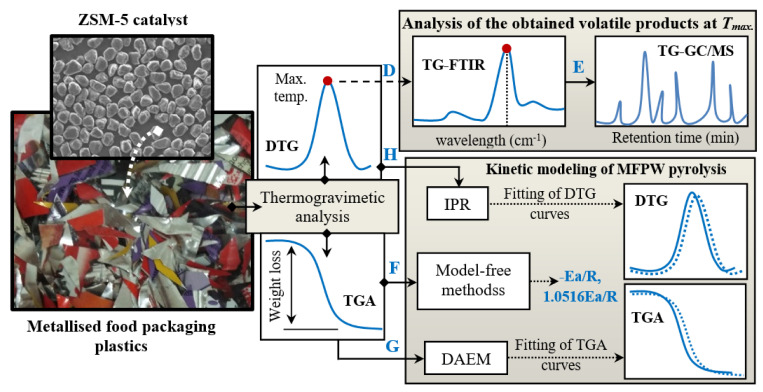
Flowchart of the experiments and analysis in the current work.

Figure 1 shows the layout of the experiments of the present work. As shown in the layout, the experiments were developed in five steps: (a) study of the thermal decomposition of the samples using thermogravimetric analysis (TGA-DTG), (b) examination of the chemical structure of the obtained volatile compounds using FTIR and gas chromatography–mass spectrometry (GC/MS) analysis at the maximum temperature, (c) modelling the pyrolysis kinetic of MFPW using the model-free methods, and (d) simulation of TGA and DTG plots using DAEM and IPR, respectively. These stages with their conditions are illustrated in the next sections.

### 2.3. Thermogravimetric Experiments

First, the milled sample was mixed with ZSM-5 Zeolite catalyst of different loads (10, 30, and 50 wt.%). Afterwards, the thermogravimetric analyzer (TGA; model: STA449 F3; NETZSCH, Selb, Germany) was used to pyrolyze 8–10 mg MFPW samples in nitrogen (N_2_) ambient with flow rate of 60 mL min^−1^. The pyrolysis temperature was derived from room temperature up to 900 °C at different heating rates of 5, 10, 15, 20, 25, and 30 °C min^−1^. The TGA results in terms of mass loss were recorded using the TGA analyzer and Pyrys software-V8, while the DTG curves were obtained through derivation of TGA measurements.

### 2.4. Chemical Analysis of the Obtained Volatile Products

TG-FTIR analyzer was used to observe the functional groups and chemical structure of the volatile products obtained from TGA analysis at the maximum decomposition peaks in the scope from 300 to 400 °C representing the main decomposition regions. Additionally, these synthesized chemical compounds and the non-condensable gases were identified and quantified using the thermogravimetry-gas chromatography–mass spectrometry (TG–GC–MS, Thermo Scientific ISQ™ single quadrupole GC–MS). The micro-GC and GC–MS analyses (Agilent, Santa Clara, CA, USA) were carried out using Automation Autoinjector™ unit (to collect the gases) connected to TGA analyzer’s outlet, in the range of 30–600 m/s. The micro-GC–MS analysis was performed with specific column setting (Argon ≥ 99.999%, 20 psi, 100 °C, and 120 s), pump time (20 s), inject time (30 ms), TCD temperature (75 °C), and injector temperature (90 °C) [30].

### 2.5. Pyrolysis Kinetics of MFPW and Simulation of TGA-DTG Curves

Model-free methods were used to determine the pyrolysis kinetic parameters of MFPW as a single reaction without any more assumptions, in particular, activation energy (Ea), using Friedman method, Flynn–Wall–Ozawa method, and Kissinger–Akahira–Sunose method. Ea can be calculated from the slope of these relationships using Equations (1)–(4) and all formulas of these techniques are shown in Table 1. DAEM was used to calculate both activation energy and pre-exponential factor more accurately, thus simulating TGA curves using Equation (5). Average Ea was received from free-methods and initial guess for the minimum running time was made, thus improving the accuracy of the results. Meanwhile, the parameters needed to plot the DTG curves can be determined using IPR and Equation (6). In order to determine the optimal parameters (Ei, Ai, and Ci) that can achieve the minimum deviation between DTG experimental data and calculated data, the algorithm code supported with the gradient-based minimization function fmincon was built using MATLAB® software 2020 for that purpose. Finally, the deviations (Dev.%) between the developed models to simulate the TGA-DTG data and experimental data were calculated using Equation (7). All parameters used in the specified equations are described in Table 2.

## 3. Results and Discussion

### 3.1. TGA-DTG Analysis

**Figure 2:**
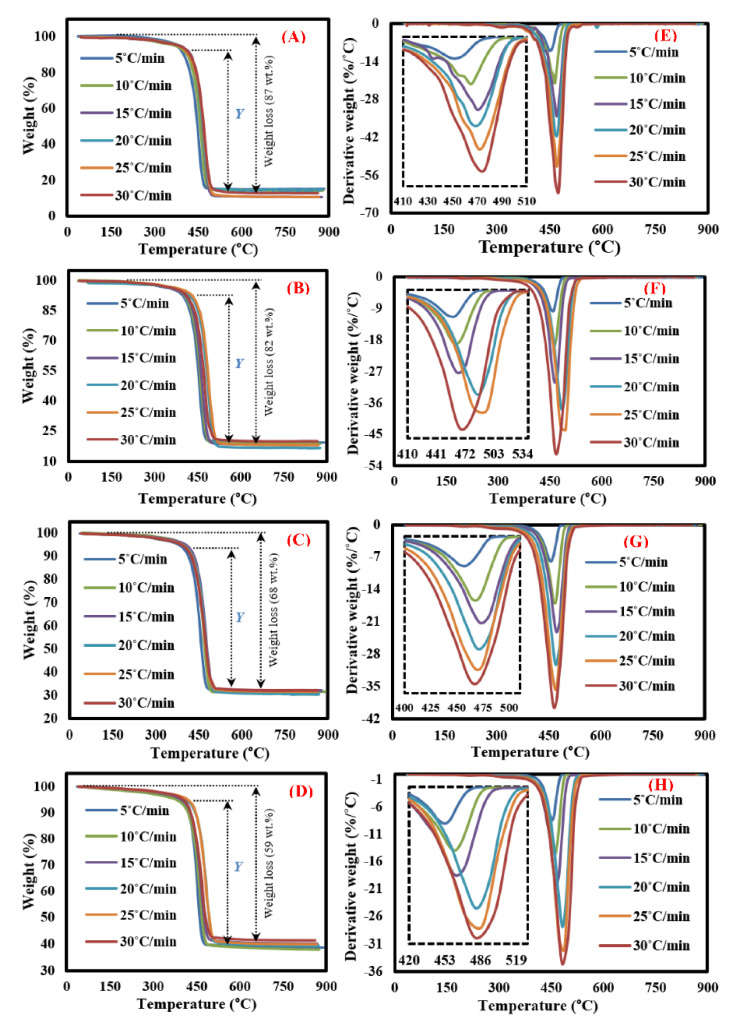
(**A**–**D**) TGA analysis and (**E**–**H**) DTG analysis of metallized food packaging plastics waste (MFPW) loaded with 0, 10, 30, and 50 wt.% at different heating rates.

TGA-DTG curves of MFPW resulting from TGA experimental measurements are displayed in Figure 2. As shown in the TGA results (Figure 2A–D), all curves have similar features, which can be described in three main decomposition phases. The first decomposition phase up to 200 °C with smaller weight loss is estimated at 0.4–1 wt.% (depending on heating rates: 5–30 °C/min) because of moisture evaporation. The second phase up to 420 °C refers to heat penetration between the layers of the decomposed MFPW sample and disassembling of their layers into two main components: polymer and Al fractions [19]. Meanwhile, the third phase (Y) can be described as a major decomposition reaction zone up to 540 °C with high weight loss due to the thermal degradation of organic components and films in the tested samples (PET, LDPE, and EVA). However, the last phase was described as a minor degradation zone like the first phase. It appears due to char devolatilization/decomposition and aluminum fraction residue [19,28]. It is clear from the experimental TGA data that increasing amount of the catalyst leads to a significant increase in the thermal resistance of the decomposed samples in terms of total weight loss, which was estimated at 87% (0 wt.%), 82% (10 wt.%), 68% (30 wt.%), and 59% (50 wt.%); this is due to the fact that the pyrolysis process is not able to decompose ZSM-5 Zeolite catalyst and leaves it as a residue, and therefore, it must be removed from the calculated TGA experimental data to obtain accurate results [33,34]. Having removed the catalyst’s weight from the calculation, it was noted that adding of catalyst did not affect the weight loss in the major decomposition region (which was estimated > 70 wt.%) and other features of the TGA curves with weight loss: 87 (0 wt.%), 90.2 (10 wt.%), 88.4 (30 wt.%), and 88.5 (50 wt.%) wt.%. The DTG curves (Figure 2E–H) show only one strong sharp decomposition peak in the range of 420–540 °C for all MFPW samples, even after changing the concentration of ZSM-5 Zeolite and heating rates of the thermal reaction, and these results agree with TGA results. However, as heating rates increased in all MFPW samples, the intensity of this single peak increased gradually with a small shifting in decomposition temperatures, due to generation of more heat flux, hence facilitating the heat exchange between the outer surroundings of the pyrolyzed sample and its internal moroclaur followed by achievement of full decomposition of all MFPW components in shorter degradation time [29,35].

### 3.2. Chemical Analysis of the Obtained Volatile Products

**Figure 3:**
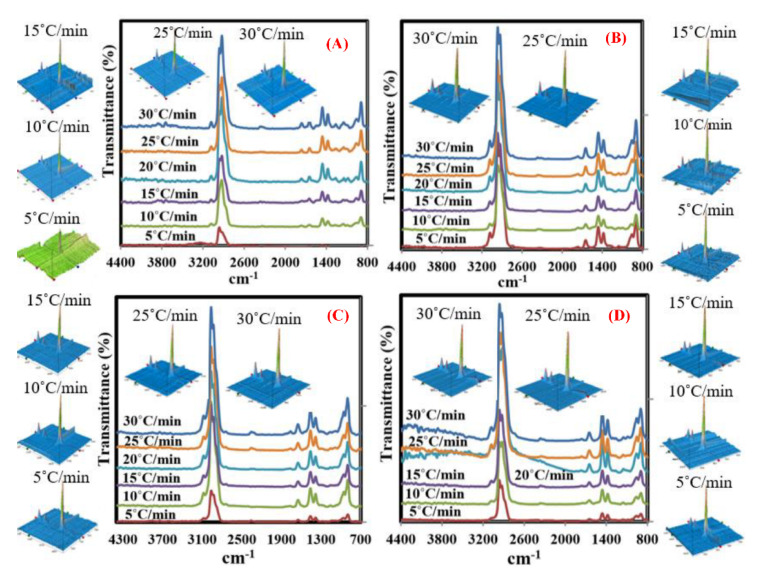
The 2–3D FTIR analysis of the decomposed ZSM-5/MFPW samples with concentration (**A**–**D**) 0, 10, 30, and 50 wt.%, at different heating rates (5–30 °C/min).

Figure 3 shows 2–3D FTIR spectra of the obtained volatile products resulting from FTIR coupled with TG at 448–476 °C (based on the DTG results) and 5–30 °C/min. In case of three catalyst samples (0 wt.%), only one strong peak was noticed at 2964 cm^−1^ at the lowest heating rate (5 °C/min) referring to methane and carboxylic acid residues. Once the heating rate increased, two other peaks appeared at 900 cm^−1^ (C-O-C stretching) and 1400 cm^−1^ (–CH_2_– bending). It was observed that the intensity of these peaks increased by increasing heating rates, especially 2964 cm^−1^, which means that the amount of the flammable compounds is directly proportional to heating rates. This is because the heating rates generating bigger heat flux are able to decompose the outer polymer layers, then penetrate to the layers below and decompose the complex organic molecules of the inner layers into methane and carboxylic acid residue compounds [19,20,36]. In case of ZSM-5/MFPW samples, the same functional groups were observed in these samples even when ZSM-5 concentration was increased, however, the absorbance of –CH_2_– bending and methane increased significantly, especially at the highest concentration of catalyst (50 wt.%) and heating rates (25 and 30 °C/min), because the unstable hydrocarbons were combined together in the polyolefins to form bigger number of flammable compounds and oil [37,38].

On the other hand, FITR 3D spectra show that the thermo-chemical reaction became very smooth and majority of disturbance peaks disappeared with increase in the heating rate of the reaction and concentration of catalyst, hence indicating that the entire plastic layers had decomposed thermally into volatile products. In order to determine and quantify the obtained products, GC-MS measurements were used in the next section as a function of heating rate and catalyst concentration.

### 3.3. Chemical Analysis of the Synthesized Chemical Compounds Using GC–MS

**Figure 4:**
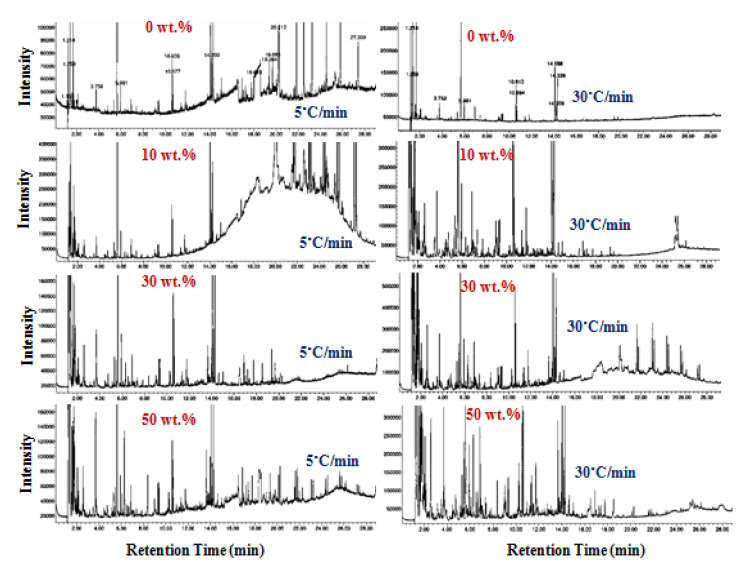
GC–MS analysis of the decomposed MFPW compounds at different loading of catalysts and different heating rates.

GC–MS measurements were carried out on the decomposed ZSM-5/MFPW samples at the lowest (5 °C/min) and the highest heating rate (30 °C/min) for each batch, where these heating rates gave the lowest and highest absorbance of –CH_2_– bending and methane functional groups, based on FTIR results mentioned in the above section. Figure 4 shows GC–MS analysis of the synthesized volatile compounds produced from the pyrolyzed MFPW samples at the lowest and the highest heating rate of 5 and 30 °C/min, while the definitions of these compounds and their respective peak areas are shown in Tables S1 and S2 in supplementary information section.

As shown in GC–MS analysis, at the lowest heating rate (5 °C/min), Propene, 1-Propene, 2-methyl-, Pentane, 2,4-Dimethyl-1-heptene compounds were the most abundant pyrolysis compounds in the released volatile products from the sample free of ZSM-5. As the concentration of ZSM-5 increased in the reaction, the intensity of Propene was not affected (7.4 wt.%), while the peak area of 2-methyl- (from 7.4% to 14.9%) and Pentane compounds increased (from 11.8% to 12.8%). Meanwhile, the peak area of 2,4-Dimethyl-1-heptene decreased significantly from 34.57% to 12.95%. In additionally, other compounds appeared at high concentration of ZSM-5 (50 wt.%), e.g., Toluene (4.31%), p-Xylene (3.82%), and Benzene, 1-ethyl-2-methyl- (1.89%), and these compounds are the main components of oil pyrolysis [39,40]. At the highest heating rate (30 °C/min), the observed GC–MS compounds had almost the same trend as at 5 °C/min, in particular, Propene (7.6–6.9%), 1-Propene, 2-methyl- (4.23–14.95 %), Pentane (12.09–15.02%), 1-Pentene, 2-methyl- (7.81–15.02%), and 2,4-Dimethyl-1-heptene (38.39–14.71%), in addition to other flammable liquid peaks, such as Benzene, 1,3-dimethyl- (2.46%), and Hexane, 3-ethyl-(3.34%) [41]. Generally, strong presence of Benzene, Hexane, Toluene compounds in large yield indicates that the paraffin wax pyrolysis product has been converted into light hydrocarbons in the form of light oil (bio-crude) [42]. In addition, strong presence of other compounds generated from the catalytic pyrolysis of ZSM-5 were typical energy products. Finally, these compounds can be used in several applications, such as chemical production, fungicide propiconazole, fuel, cleaners, pharmaceuticals, etc., [43,44].

### 3.4. Kinetic Analysis of Catalytic Pyrolysis of MFPW

Kinetics of MFPW catalytic pyrolysis using ZSM-5 Zeolite catalyst with different concentrations (10, 30, and 50 wt.%) were presented and analyzed in five phases: (a) estimation of activation energy for the whole catalytic pyrolysis process of MFPW, using Kissinger method, (b) estimation of activation energy at every conversion rate during the catalytic pyrolysis process of MFPW, using FWO, KAS, and Friedman models, (c) fitting of TGA curves using DAEM model, and (d) fitting of TGA data using IPR model.

#### 3.4.1. Evaluation of Activation Energy for the Entire MFPW Catalytic Pyrolysis

**Figure 5:**
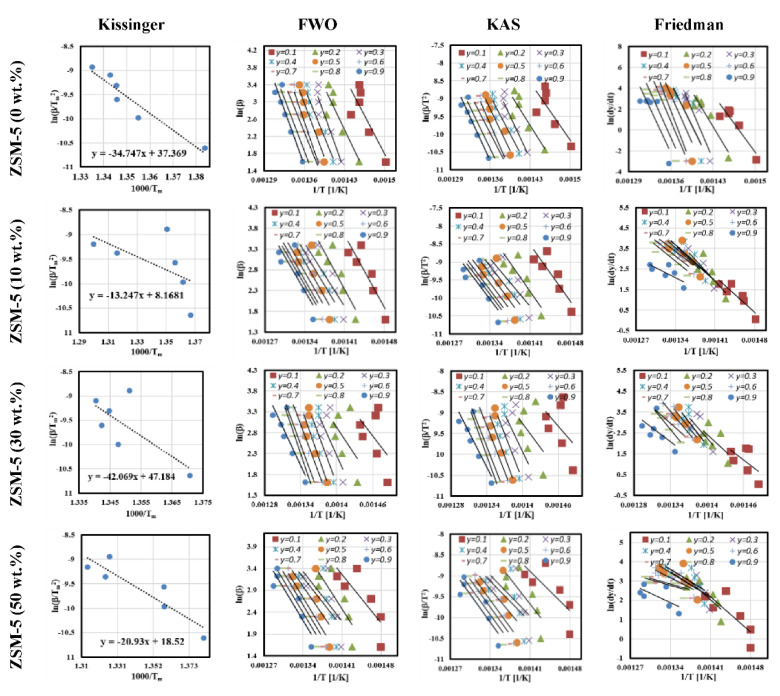
Plots of isoconversional and model-free methods curves.

Figure 5 shows the fitted ln(*β*/$T_{m}^{2}$) versus *1/T* curves using Kissinger approach for all heating rates. These curves were used to calculate the whole activation energy for the whole catalytic pyrolysis process of MFPW, where the slope of these fitted curves can be expressed as −Ea/R (R = 8.31 JK^−1^ mol^−1^). Based on the calculated terms, Ea was estimated at 289 kJ/mol (0 wt.%), 110 kJ/mol (10 wt.%), 350 kJ/mol (30 wt.%), and 174 kJ/mol (50 wt.%). As shown in the results, 30 wt.% of catalyst gave the highest Ea with increase of 21%, when compared with the free catalyst sample.

#### 3.4.2. Estimation of Activation Energies for Each Conversion Zone

Activation energies as a function conversion zone (from 10% to 90%) were calculated similarly to the above section by fitting ln(β/T2) versus 1000/T, lnβ versus 1/T, and ln(dx/dt) versus 1/T curves, and then determining the slope of each curve expressed as −Ea/R (KAS and Friedman) and 1.0516Ea/R (FWO), as shown in Figure 5. As shown in the curves, the fitted lines are straight and parallel mostly in the whole conversion zone, especially KAS and FWO plots for all loading of ZSM-5. Although the lines plotted using the Friedman model were straight, these lines were distributed randomly, especially with increase in the concentration of ZSM-5 at lower and higher conversion, which means that FWO and KAS models are more appropriate to model the reaction mechanism of MFPW in the entire conversion region.

**Figure 6:**
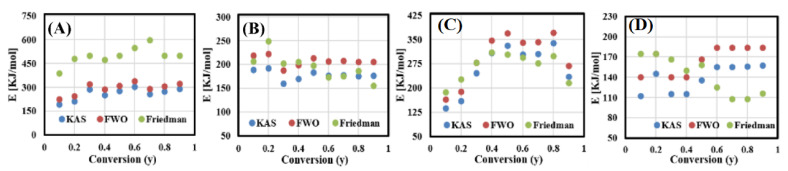
The apparent activation energy-conversion curves for (**A**) MFPW and (**B**–**D**) 10, 30, 50 wt.% of ZSM-5.

Figure 6 and Table 3 show the activation energies at all conversion rates in the range of 10–100% calculated using KAS, FWO, and Friedman methods. It is clear that KAS and FWO manifested almost the same trend of Ea in all conversion zones, while Friedman gave some variation in Ea values compared to other methods (KAS and FWO), especially in MFPW and ZSM-5 (50%)/MFPW samples. Additionally, MFPW and ZSM-5 (30%)/MFPW samples manifested the maximum Ea within the range of 0.3–0.8 due to simultaneous contacting of unstable radicals [45], while ZSM-5 (10 and 50 wt.%)/MFPW samples had lower Ea, and these results agree with Kissinger results presented in the above section. Based on these results, the model-free approaches are reliable to describe the reaction mechanism of catalytic pyrolysis of MFPW in the main decomposition region (0.3–0.8).

#### 3.4.3. Fitting of TGA Data Using DAEM

**Figure 7:**
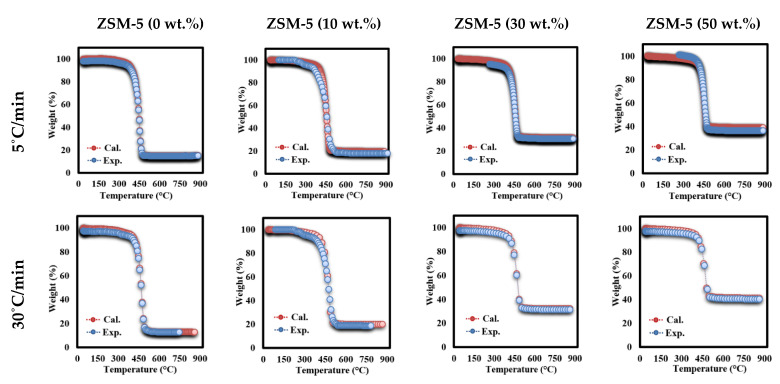
Fitting TGA experimental and calculated data for MFPW and ZSM-5 (30%)/MFPW samples at 5 and 30 °C/min.

Figure 7 shows the TGA experimental curves and the fitted TGA curves for MFPW and ZSM-5/MFPW samples at 5 °C/min (lowest heating rate) and 30 °C/min (highest heating rate) received while using Equation (5). It is clear that the fitting curves and TGA experimental data match completely the deviation <1 (calculated using Equation (7)) for both MFPW and ZSM-5/MFPW samples at 5 and 30 °C/min. These results prove that DAEM approach can be used to model TGA experimental curves of ZSM-5/MFPW samples at different heating rates and catalyst concentrations. Finally, the activation energies (E) and pre-exponential factor (A) for the pseudo components for all the sets of ZSM-5/MFPW samples calculated using DAEM are summarized in Table 4. As shown in the Table, each set has two values of E (E1 and E2) and A (A1 and A2), where E1 and E2 represent energies at weak and strong decomposition peaks, respectively, and are similar for A1 and A2. All these parameters were obtained from the developed model coupled with an optimization algorithm, and these parameters need to fit to the TGA curves with minimum deviation [46].

**Figure 8:**
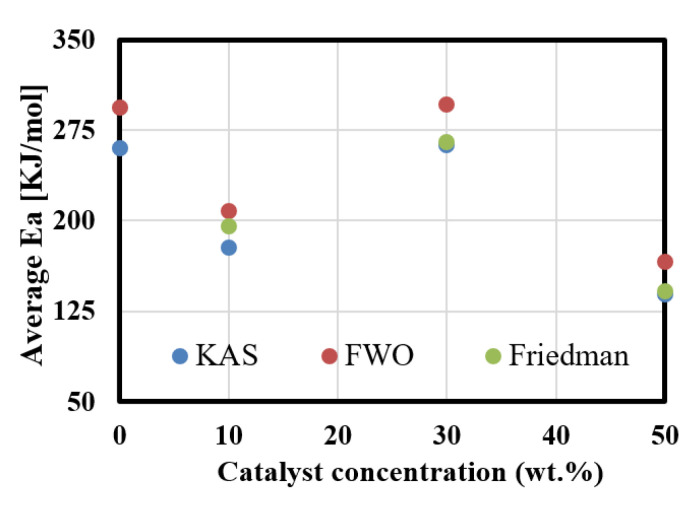
Effect of catalyst concentration on the apparent activation energy.

Finally, the relationship between the average activation energy (calculated using KAS, FWO, and Friedman methods) and the Zeolite loading is shown in Figure 8. As shown in the figure, the calculated energy using KAS, FWO, and Friedman methods almost matched together. Moreover, the maximum activation energy can be achieved at 30 wt.% of catalyst, while 50 wt.% of the catalyst has been shown to be of the lowest value with an estimated reduction in 45% due to the conversion of small feedstocks from the feedstock to light hydrocarbons as shown in the GC-MS results.

#### 3.4.4. Fitting of DTG Data Using IPR

**Figure 9:**
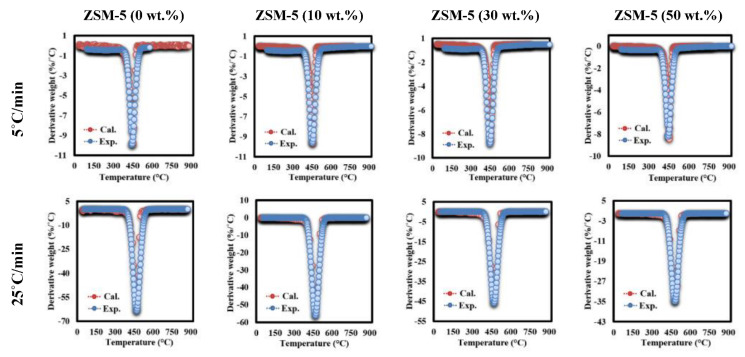
Fitting of DTG experimental and calculated data for MFPW and ZSM-5 (30%)/MFPW samples at 5 and 30 °C/min.

As it has been mentioned before, MFPW is composed of more than two pseudo elements, including PET, LDPE, EVA, and Al. However, the DTG curves of MFPW and ZSM-5/MFPW samples showed only one decomposition peak resulting from simultaneous degradation of all pseudo-organic elements together in the form of single reaction. In this section, the IPR approach was used to plot the experimental DTG data using Equation (6). Figure 9 shows the DTG experimental curves and calculated curves of MFPW and ZSM-5/MFPW samples at 5 and 30 °C/min. As shown in the figures, both DTG experimental and calculated data are fully applicable for all samples with a very small deviation <1, which means that IPR model is a promising approach to calculate kinetic parameters and to plot DTG curves of MFPW and ZSM-5/MFPW samples with smaller deviation. Therefore, the catalytic pyrolysis process using 50% of ZSM-5 Zeolite catalyst is a promising tool that could be applied for MFPW valorization and upgrading of their volatile compounds into light hydrocarbons.

## 4. Conclusions

In the present research, the catalytic pyrolysis behavior of a mixture of metalized food packaging plastics waste (MFPW) and its kinetic parameters with ZSM-5 Zeolite catalyst were investigated using the TG-FTIR-GC-MS measurements. The TG-FTIR-GC-MS experimental results and catalytic pyrolysis kinetic analysis of MFPW revealed the following:

- TGA measurements were employed to determine the effect of ZSM-5 addition and its concentrations on thermal decomposition of MFPW sample, thus revealing that TGA and DTG profiles were not affected by the catalyst with a total weight loss estimated at 87–90 wt.%.
- FTIR results showed that at the maximum degeneration temperatures, methane and carboxylic acid residues, C-O-C stretching and –CH_2_– bending are the main volatile components and their intensity increased with increase in ZSM-5 concentration and heating rate.
- GC-MS analysis showed that, at 50 wt.% of ZSM-5, the pyrolyzed MFPW sample was very rich in volatile and flammable compounds (e.g., benzene, hexane and toluene), which indicates that the catalytic pyrolysis process can be used to convert paraffin wax resulting from pyrolysis of MFPW into bio-crude and light hydrocarbons (petroleum oil).
- The kinetic models of pyrolysis, for which model-free methods were applied, revealed that the maximum activation energies can be achieved at 30 wt.% of catalyst and estimated at 263 kJ mol−1 (KAS) and 296 kJ mol-1 (FWO).
- DAEM and IPR were successful for simultaneous fitting of the TGA and DTG experimental data with deviations below <1. In addition, the pre-exponential factor was calculated using DAEM and IPR.

According to the mentioned results, the presence of catalyst during the reaction has a positive effect on the yield of volatile components. Additionally, the form of decomposition does not change by adding the catalysts; decomposition is maintained at single reaction peak, which confirms that model-free approaches can be classified as the best choice to simulate pyrolysis kinetics in presence and absence of the catalyst. In addition, DAEM and IPR models are highly recommended to simulate the catalytic pyrolysis of MFPWs with high prediction accuracy.

## Figures and Tables

**Table 1:** Methods used to determine kinetic parameters for pyrolysis of metallised food packaging plastics waste (MFPW) [31,32].

Equation No.	Method	Expressions (1)–(7)	Plots	Slope Value
(1)	Kissinger	$\ln\left(\frac{\beta }{T_{m}^{2}}\right)\;$ = $\ln\left(\frac{AR}{E_{a}}\right)\;$ − $\frac{E_{a}}{RT}$	ln(*β*/$T_{m}^{2}$) versus *1/T*	−Ea/R
(2)	Kissinger–Akahira–Sunose	$\ln\left(\frac{\beta }{T^{2}}\right)\;$ = $\ln\left(\frac{AR}{Eag\left(\alpha \right)}\right)\;$ − $\frac{-Ea}{RT}$	ln(*β*/*T*^2^) versus *1/T*	−Ea/R
(3)	Flynn–Wall–Ozawa	lnβ = $\left(\frac{\mathrm{lnA}Ea}{Rg\alpha }\right)\;$– 5.335 – $\frac{1.0516Ea}{RT}$	ln*β* versus *1/T*	−1.0516 Ea/R
(4)	Friedman	$\ln\left(\frac{\beta dy}{dT}\right)$ = $\ln\left(Af\left(y\right)\right)\left(\frac{-Ea}{RT}\right)$	ln*(dy/dt)* versus *1/T*	−Ea/R
(5)	DAEM	$\ln\left(\frac{\beta }{T^{2}}\right)\;$ = $\ln\left(\frac{AR}{Ea}\right)+0.6075\;$ − $\frac{Ea}{RT}$		
(6)	IPR	${\frac{dm}{dt}}^{calc}=-\left(m_{0}-m\right)\overset{3}{\underset{i=1}{\sum }}C_{i}\frac{dX_{i}}{dt}\;$		
(7)	Dev.(%)	Dev.(%) = $\frac{100\sqrt{F.O._{DTG}\left(Z-N\right)}}{\max\left(\left|dm/dt\right|\right)}$		

**Table 2:** Parameters of the used models [31,32].

Parameters	Definition
*β*	Heating rate
$E_{a}$	Activation energy
R	Gas constant (J mol^−1^ K^−1^)
A	Pre-exponential factor (min^−1^)
T	Temperature
*C_i_*	Mass fraction of each of three subcomponents
*dm/dt*	Rate of mass loss

**Table 3:** The calculated apparent activation energy at different conversion.

*Y*	*KAS* (KJ/mol)				*FWO* (KJ/mol)				*Friedman* (KJ/mol)			
	ZSM-5 (wt.%)				ZSM-5 (wt.%)				ZSM-5 (wt.%)			
	0%	10%	30%	50%	0%	10%	30%	50%	0%	10%	30%	50%
0.1	194	189	138	112	224	219	165	140	389	206	187	175
0.2	211	192	160	146	243	222	189	175	479	249	226	175
0.3	286	159	246	115	321	188	279	140	499	202	278	166
0.4	252	170	309	115	286	199	347	140	474	205	310	150
0.5	277	184	331	136	311	214	369	166	499	198	303	158
0.6	304	177	304	155	340	207	340	184	549	173	293	125
0.7	257	177	305	155	291	207	342	184	599	176	277	108
0.8	273	176	338	156	306	205	371	184	499	187	298	108
0.9	290	176	235	157	323	206	268	184	499	156	215	116
Avg.	260	178	263	139	294	208	296	166	498	195	265	142

**Table 4:** The pyrolysis characteristic parameters for MFPW and ZSM-5 (30%)/MFPW samples at 5 and 30 °C/min.

	DAEM				IPR			
	ZSM-5 (wt.%)				ZSM-5 (wt.%)			
	0%	10%	30%	50%	0%	10%	30%	50%
E1	293.36	173.302	247.577	152.431	231.754	136.908	195.585	120.420
A1	1.49 × 10^10^	2.99 × 10^13^	3.74 × 10^22^	3.92 × 10^10^	2.49 × 10^6^	4.99 × 10^9^	6.25 × 10^18^	6.55 × 10^6^
E2	363.766	214.895	306.996	189.014	343.557	202.9563	289.940	178.513
A2	1.64 × 10^10^	3.28 × 10^13^	4.11 × 10^22^	4.31 × 10^10^	6.27 × 10^6^	1.26 × 10^10^	1.57 × 10^19^	1.65 × 10^7^

## Data Availability

The data presented in this study are available on request from the corresponding author.

## Supplementary Materials

The following are available online at https://www.mdpi.com/2073-4360/13/5/702/s1, Table S1: GC-MS compounds generated at 5 °C/min, Table S2: GC-MS compounds generated at 30 °C/min.
