# Supplementary material for: Catalytic Pyrolysis Kinetic Behavior and TG-FTIR-GC–MS Analysis of Metallized Food Packaging Plastics with Different Concentrations of ZSM-5 Zeolite Catalyst
Source: Polymers (Basel). 2021 Feb 26;13(5):702. doi: 10.3390/polym13050702 (PMC7956265; doi:10.3390/polym13050702)
Supplement: Supplementary file 1 [file polymers-13-00702-s001.pdf]

Table S1. GC-MS compounds generated at 5 °C/min.

0 wt.%			10 wt.%			30 wt.%			50 wt.%		
**Time (min.)**	**GC Compounds**	**Area (%)**	**Time (min.)**	**GC Compounds**	**Area (%)**	**Time (min.)**	**GC Compounds**	**Area (%)**	**Time (min.)**	**GC Compounds**	**Area (%)**
**1.214**	Propene	**7.42**	**1.200**	Propene	**1.26**	1.200	Propene	**5.47**	**1.20**	Propene	**7.38**
**1.259**	1-Propene, 2-methyl-	**7.34**	**1.245**	1-Propene, 2-methyl-	**2.41**	1.245	1-Propene, 2-methyl-	**10.54**	**1.246**	1-Propene, 2-methyl-	**14.90**
**1.395**	Pentane	**11.81**	**1.381**	Pentane	**3.79**	1.375	Pentane	**13.17**	**1.375**	Pentane	**12.83**
**1.679**	1-Pentene, 2-methyl-	**7.25**	**1.426**	2-Butene, 2-methyl-	**0.93**	1.420	2-Butene, 2-methyl-	**4.65**	**1.420**	2-Butene, 2-methyl-	**5.71**
**3.762**	Heptane, 4-methyl-	**1.92**	**1.659**	1-Pentene, 2-methyl-	**1.57**	1.562	1H-Pyrazole, 4,5-dihydro-5-methyl-	**2.25**	**1.569**	1H-Pyrazole, 4,5-dihydro-5-methyl-	**2.44**
**5.671**	2,4-Dimethyl-1-heptene	**34.57**	**1.750**	2-Butene, 2,3-dimethyl-	**1.27**	1.653	1-Pentene, 2-methyl-	**3.08**	**1.653**	1-Pentene, 2-methyl-	**2.33**
**5.981**	Cyclohexane, 1,3,5-trimethyl-, alpha.,3.alpha.,5.beta.)-	**2.03**	**5.605**	2,4-Dimethyl-1-heptene	**9.11**	1.743	2-Pentene, 3-methyl-, €-	**6.11**	**1.744**	2-Pentene, 2-methyl-	**5.73**
**6.925**	5-Aminoisoxazole Spermine	**1.55**	**10.560**	Ethanone, 1-cyclopentyl-	**1.32**	1.828	2-Pentene, 3-methyl-, (Z)-	**1.59**	**1.828**	2-Pentene, 3-methyl-, €-	**1.46**
**10.613**	Ethanone, 1-cyclopentyl-	**3.57**	**10.638**	1R,2c,3t,4t-Tetramethyl-cyclohexan	**0.97**	2.584	1,3-Pentadiene, 2,3-dimethyl-	**2.28**	**2.585**	1,4-Hexadiene, 5-methyl-	**2.31**
**10.684**	N-Methylallylamine	**2.49**	**14.047**	Heptane, 2-methyl-3-methylene-	**2.50**	3.697	Heptane, 4-methyl-	**2.97**	**3.697**	Toluene	**4.31**
**14.086**	2-Undecene, 4-methyl-	**7.81**	**14.163**	Cyclopentane, (2-methylbutyl)-	**1.00**	5.605	2,4-Dimethyl-1-heptene	**22.29**	**5.605**	2,4-Dimethyl-1-heptene	**12.95**
**14.203**	2-Decene, 7-methyl-, (Z)-	**2.67**	**14.280**	Hexane, 2,3,4-trimethyl-	**2.12**	5.922	Cyclohexane, 1,3,5-trimethyl-, (1. Alpha.,3.alpha.,5. beta.)-	**2.30**	**5.922**	Cyclohexane, 1,3,5-trimethyl- ,alpha.,3.alpha.,5.beta.) -	**1.56**
**14.326**	Dichloroacetic acid, 6-ethyl-3-octyl ester	**6.92**	**21.628**	Fumaric acid, nonyl 2,3,6-trichlorophenyl ester	**7.42**	6.297	p-Xylene	**1.59**	**6.285**	p-Xylene	**3.82**
**15.063**	Cyclododecanemethanol	**1.31**	**21.732**	Cyclodecasiloxane, eicosamethyl-	**2.28**	10.567	1-Undecene, 7-methyl-	**3.17**	**8.374**	Benzene, 1-ethyl-2-methyl-	**1.89**

16.920	Acetic acid, trifluoro-, 3,7-dimethyloctyl ester	1.35	22.579	4,4'-(Hexafluoroisopropylidene)diphenol	4.14	10.638	Ethanone, 1-cyclopentyl-	2.39	10.560	1-Undecene, 7-methyl-	2.08
			23.051	4-Thiazolemethanol, 2-(4-chlorophenyl)-	7.75	14.047	2-Acetylcyclopentanone	6.28	10.638	1R,2c,3t,4t-Tetramethyl-cyclohexan	1.58
			23.219	Cyclodecasiloxane, eicosamethyl-	4.65	14.163	Cyclopentane, (2-methylbutyl)-	2.34	13.646	1H-1,2,4-Triazole, 3-(2-methylpropyl)-	1.61
			24.358	Fumaric acid, nonyl 2,3,6-trichlorophenyl ester	6.94	14.286	Cyanamide, dibutyl-	5.61	13.982	Cyclohexanone, 3,3,5-trimethyl-	1.71
			24.513	Cyclononasiloxane, octadecamethyl-	4.94	16.498	Octasiloxane, 1,1,3,3,5,5,7,7,9,11,11,13,13,15,15-hexadecamethyl-	1.92	14.047	2-Acetylcyclopentanone	4.28
			24.655	Pyrazole-4-carboxamide, 3-(1-adamantyl)-1-phenyl-	2.67				14.163	2-Undecene, 4-methyl-	1.63
			25.606	4-Nitro-4'-chlorodiphenylsulfoxide	6.62				14.280	2-Acetylcyclopentanone	3.76
			25.768	Cyclononasiloxane, octadecamethyl-	5.53				16.499	Cyclohexane, 1,1'-(2-ethyl-1,3-propanediyl)bis-	1.57
			27.172	4-Nitro-4'-chlorodiphenylsulfoxide	5.01				20.257	1,1,1,5,7,7,7-Heptamethyl-3,3-bistrimethylsiloxy)tetrasiloxane	2.17
			27.340	Cyclodecasiloxane, eicosamethyl-	5.67						
			29.352	Pentasiloxane, dodecamethyl-	3.31						
			29.487	Hexasiloxane, tetradecamethyl-	4.83						

Table S2. GC-MS compounds generated at 30 °C/min.

0 wt.%			10 wt.%			30 wt.%			50 wt.%		
**Time (min.)**	**GC Compounds**	**Area (%)**	**Time (min.)**	**GC Compounds**	**Area (%)**	**Time (min.)**	**GC Compounds**	**Area (%)**	**Time (min.)**	**GC Compounds**	**Area (%)**
1.214	Propene	7.60	1.213	Propene	3.95	1.200	Propene	6.46	1.213	Propene	6.89
1.259	1-Propene, 2-methyl-	4.23	1.258	1-Propene, 2-methyl-	7.59	1.245	1-Propene, 2-methyl-	12.88	1.258	1-Propene, 2-methyl-	14.95
1.395	Pentane	12.09	1.394	Pentane	15.46	1.381	Pentane	12.49	1.388	Pentane	15.02

1.679	1-Pentene, 2-methyl-	7.81	1.582	2-Pentene, 4-methyl-, (Z)-	1.99	1.426	Cyclopropane, 1,2-dimethyl-, cis-	5.75	1.433	Cyclopropane, 1,2-dimethyl-, cis-	5.68
3.756	Heptane, 4-methyl-	2.12	1.672	1-Pentene, 2-methyl-	6.07	1.569	1H-Pyrazole, 4,5-dihydro-5-methyl-	2.21	1.582	Cyclopropane, 1,1,2-trimethyl-	3.17
5.671	2,4-Dimethyl-1-heptene	38.39	1.757	2-Pentene, 3-methyl-, (Z)-	3.21	1.659	1-Pentene, 2-methyl-	3.22	1.666	1-Pentene, 2-methyl-	2.42
5.981	Cyclohexane, 1,3,5-trimethyl-, alpha.,3.alpha.,5.alpha.)-	1.94	2.597	1,3-Pentadiene, 2,3-dimethyl-	2.14	1.750	2-Butene, 2,3-dimethyl-	5.58	1.757	2-Pentene, 3-methyl-, (Z)-	5.33
6.926	2-Pentanone, 3-[(acetyloxy)methyl] -3,4-dimethyl-, (.+-.)-2-	1.55	3.710	Heptane, 4-methyl-	2.34	1.834	2-Pentene, 3-methyl-, €-	1.75	1.841	2-Pentene, 3-methyl-	1.56
10.606	Ethanone, 1-cyclopentyl-	3.55	5.618	2,4-Dimethyl-1-heptene	30.38	2.067	Cyclopentene, 1-methyl-	1.43	2.074	Cyclopentene, 1-methyl-	1.67
10.677	4-Methyl-2-heptene	2.53	5.929	Cyclohexane, 1,3,5-trimethyl-, (1. Alpha.,3.alpha.,5.beta.)-	2.06	2.591	1,3-Pentadiene, 2,3-dimethyl-	2.81	2.597	2,4-Hexadiene, 2-methyl-	2.86
14.086	2-Decene, 7-methyl-, (Z)-	5.90	6.873	1-Silacyclo-3-pentene	1.52	3.703	Heptane, 4-methyl-	2.30	3.710	Heptane, 4-methyl-	3.03
14.203	2-Decene, 7-methyl-, (Z)-	1.91	10.567	2-Undecene, 4-methyl-	3.85	5.463	2-Hepten-4-one, 2-methyl-	1.60	5.463	2-Hepten-4-one, 2-methyl-	1.79
14.319	4-Decene, 3-methyl-, €-	4.78	10.638	2-Undecene, 4-methyl-	2.68	5.612	2,4-Dimethyl-1-heptene	17.61	5.612	2,4-Dimethyl-1-heptene	14.71
19.378	Cyclohexane, 1-ethyl-2-propyl-	1.43	14.047	2-Acetylcyclopentanone	5.65	5.922	Cyclohexane, 1,3,5-trimethyl- ,alpha.,3.alpha.,5.beta.)-	1.67	5.929	Cyclohexane, 1,3,5-trimethyl- ,alpha.,3.alpha.,5.beta.)-	1.57
20.290	1,1,1,5,7,7,7-Heptamethyl-3,3-bis trimethylsiloxy)tetrasiloxane	2.75	14.170	2-Acetylcyclopentanone	1.83	6.284	p-Xylene	1.55	6.291	Benzene, 1,3-dimethyl-	2.46
21.849	1,1,1,5,7,7,7-Heptamethyl-3,3-bis trimethylsiloxy)tetrasiloxane	1.43	14.286	4-Isopropyl-1,3-cyclohexanedione	4.45	6.867	1-Silacyclo-3-pentene	1.44	6.873	1-Dodecanone, 1-cyclopropyl-	1.78
			25.199	Cyclononasiloxane, octadecamethyl-	2.42	10.560	2-Undecene, 4-methyl-	2.71	10.567	2-Undecene, 4-methyl-	2.74
			25.406	Octasiloxane, 1,1,3,3,5,5,7,7,9,9,11,11,13,13,15,15-hexadecamethyl-	2.40	10.638	Ethanone, 1-cyclopentyl-	1.77	10.638	3-Dodecene, €-	1.80
						14.047	2-Acetylcyclopentanone	3.69	13.646	Oxalic acid, cyclohexyl dodecyl ester	1.60
						14.280	2,3-Dimethyl-3-heptene, (Z)-	2.92	13.982	3-Methyl-2-butenoic acid, 6-ethyl-3-octyl ester	1.56

		**20.108**	Cyclononasiloxane, octadecamethyl-	**2.41**	**14.047**	2-Acetylcyclopentanone	**4.08**
		**21.641**	1-(2-Methylbutoxy)-7-heptyl-2,2,4,	**1.53**	**14.286**	Hexane, 3-ethyl-	**3.34**
		**21.796**	Cyclodecasiloxane, eicosamethyl-	**1.35**			
		**23.058**	Dimethyl 6-methoxyquinolinate	**1.60**			
		**24.358**	Octasiloxane, 1,1,3,3,5,5,7,7,9,9,11,11,13,13,15,15-hexadecamethyl-	**1.24**			
